# Intraluminal Duplication of the Terminal Ileum with Ectopic Gastric Mucosa in an Infant: A Rare Cause of Intussusception

**DOI:** 10.1155/2020/6898795

**Published:** 2020-01-27

**Authors:** Filomena Valentina Paradiso, Laura Merli, Sara Silvaroli, Vincenzo Fiorentino, Riccardo Ricci, Lorenzo Nanni

**Affiliations:** ^1^Division of Pediatric Surgery, Fondazione Policlinico Universitario “Agostino Gemelli” IRCCS, Largo Agostino Gemelli 8, 00168 Rome, Italy; ^2^Department of Pathology, Università Cattolica del Sacro Cuore, Largo Vito 1, 00168 Rome, Italy; ^3^UOC di Anatomia Patologica, Fondazione Policlinico Universitario “A Gemelli” IRCCS, Largo A. Gemelli 8, 00168 Rome, Italy

## Abstract

Enteric duplication cysts are rare malformations mostly diagnosed before the age of two, with varied clinical presentations. Ectopic gastrointestinal epithelium can be present, and management involves surgical resection. A three-month-old girl presented with rectal bleeding due to an ileocolic intussusception. Abdominal ultrasound revealed a target sign in the right upper part of the abdomen. At hydrostatic contrast enema, an incomplete reduction of the intussusception was obtained: only a trickle of contrast material entered the terminal ileum. An exploratory laparotomy ensued with manual reduction of the intussusception. At the end of the maneuver, a soft intraluminal mass was palpated within the ileocecal valve. Thus, an ileocecal resection was performed. At histology, an intraluminal enteric duplication cyst was documented, containing ectopic gastric mucosa. Secondary intussusception should be suspected even in infants in case of abnormal findings at hydrostatic contrast enema. Intraluminal enteric duplication cysts may be a rare cause of intussusception.

## 1. Introduction

Enteric duplication cysts are a rare congenital anomaly with varied clinical presentations that require surgical resection for definitive treatment [1]; the reported incidence is 1 in 4500. The majority (80%) of these lesions is diagnosed before the age of two and can occur anywhere from the oropharynx to the anus. Enteric duplications frequently contain mucosa similar to that of their adjacent gastrointestinal location, but this is not always the case [2]. We present the case of an intussusception due to a duplication cyst containing gastric mucosa that was entirely intraluminal.

## 2. Case Presentation

**Figure 1:**
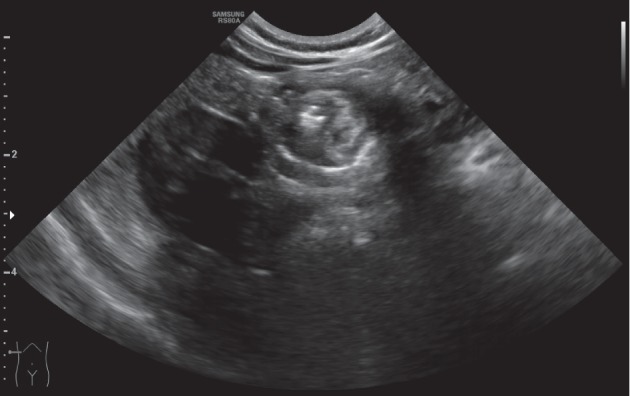
The classical target sign seen at ultrasound which prompted the hydrostatic enema.

**Figure 2:**
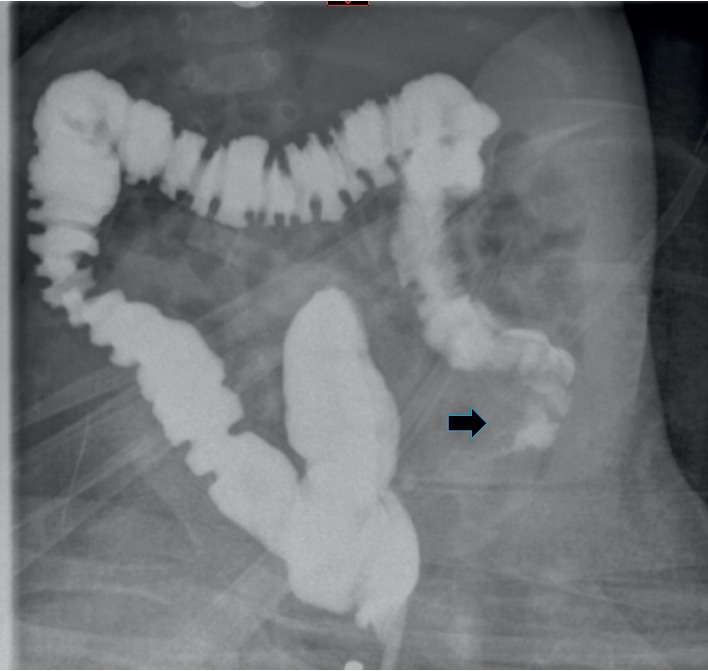
Hydrostatic enema shows complete opacification of the ascending colon, but no flooding of contrast material in the small bowel is observed. A crescent-like defect (arrow) in the cecal contour is visible.

**Figure 3:**
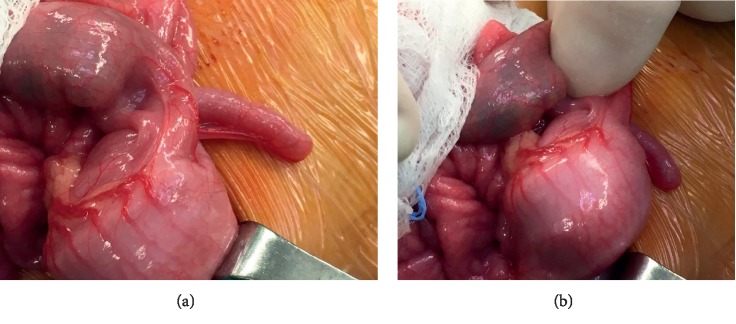
At laparotomy, a small intraluminal round-shaped mass can be palpated within the terminal ileum. The mass was detectable only when it was pushed out in the cecum.

**Figure 4:**
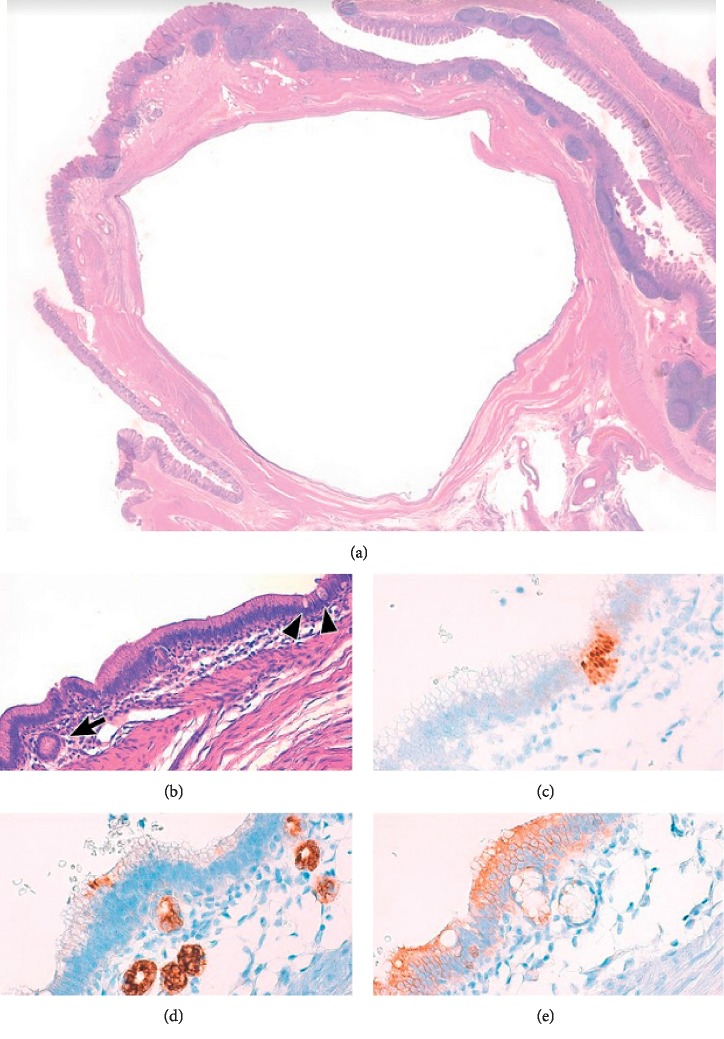
Representative images of the reported lesion. (a) Pathological examination revealed a 1.7 cm cyst located in the muscularis propria of the ileocecal valve partly disepithelized. (b) The cyst lining consisted of mucosa mostly with gastric-type features, with pyloric-type glands (arrow), and focally with intestinal features with goblet cells (arrowheads). At immunohistochemistry, CDX2, MUC6, and MUC5AC highlighted residual intestinal epithelium (c), pyloric-type glands (d), and gastric-type foveolar surface epithelium (e), respectively (original magnification: (a) ×2; (b) ×200; (c)–(e) ×400).

A full-term three-month-old girl presented at the emergency room for rectal bleeding. Her perinatal history was unremarkable, and the clinical evaluation was normal. Abdominal ultrasound revealed a target sign in the right upper quadrant with a hypoechoic formation 1.6 cm in diameter (Figure 1). Laboratory evaluation revealed normal C-reactive-protein (0.04 mg/dL; normal ≤0.5 mg/dL), normal haemoglobin (12.6 g/dL; normal 12.0–15.0 g/dL), and normal haematocrit (37.0%; normal 36–46%), erythrocytes (4.78 × 10^12/L^ normal 4.50–5.50 × 10^12/L^), and leukocytes (6.15 × 10^9/L^; normal 4.00–10.00 × 10^9/L^). At hydrostatic contrast enema, the cecum was visualized but flooding of the terminal ileum with contrast material was not obtained (Figure 2). After parent's informed consent, the patient was taken to the operating room and a right-sided transverse laparotomy was performed. The ileocecal region was exteriorized, and the intussusception was confirmed and manually reduced. A spherical soft mass was palpated within the lumen of the terminal ileum (Figure 3). Ileocecal resection and primary anastomosis was performed, incorporating the mass. Postoperatively, the baby did well and was discharged on postoperative day 6. On gross examination, the resected specimen had a length of 4.7 cm and showed an ileocecal valve with a spherical cyst, sized 1.7 cm, centered in the muscularis propria; no communication with the intestine lumen was seen. Histology of the cyst revealed a mucosal lining mostly with gastric-type features, with pyloric-type glands and surface foveolar-type epithelium (highlighted by MUC6 and MUC5AC immunoreactivity, respectively); focally, intestinal-type epithelial features characterized by the presence of goblet cells and CDX2 nuclear expression were also present (Figure 4). At 3-month follow-up, the child is doing well, tolerating feeds, and meeting all developmental milestones.

## 3. Discussion

Duplications of the small bowel are most commonly encountered. They are generally located on the mesenteric side of the intestine as opposed to Meckel's diverticula which occur on the antimesenteric side.

The majority of duplication cysts is diagnosed in the first 2 years of life. They present in a variety of ways depending on their size, location, adjacencies, and whether they contain heterotopic gastric mucosa. In some cases, intestinal or respiratory mucosa, but also annular or an ectopic pancreas, was associated [3]. Due to their mesenteric location, duplication cysts frequently share the muscular wall and blood supply with the adjacent intestine. Enteric duplication cysts have been classified into three subtypes: saccular (spherical), tubular, and small intramural [4]. The saccular type is the most common type which usually does not communicate with the lumen and is often asymptomatic. Tubular cysts tend to occur more commonly in the colon [4]. Small intramural cysts often occur near or at the ileocecal valve and protrude into the bowel lumen. Multiple theories have arisen to explain the occurrence of enteric duplication, but no single theory can account for all the known variants. Etiopathogenetic theories include disorders of the embryonal development of the digestive system similar to the persistence of embryonic diverticula, partial or abortive twinning, vascular malformations, a split notochord, and aberrant recanalization following the solid phase of fetal enteric mucosal development. Finally, it is known that there are significant environmental stresses on the fetus at different times [2, 3].

Signs and symptoms may vary though they generally present with abdominal pain and/or the finding of a mass. Small cystic duplications can result in localized volvulus or they can act as a lead point for small bowel intussusception. In 1989, Adamsbaum et al. first reported a 17-month-old boy who presented with a history of abdominal pain and vomiting due to an ileocecal straddling duplication cyst [5]. In 2016, Ladd et al. described a two-month-old girl who presented with signs and symptoms of a distal small bowel obstruction due to an intraluminal duplication cyst [6]. Finally, in 2018, Lai et al. reported a 1-year-old boy with a history of recurrent abdominal pain due to intermittent intussusceptions caused by a duplication cyst [7]. In all the reported cases, surgical treatment consisted in an ileocecal (IC) resection. There are few and discussed data regarding the consequences of the loss of the IC valve in children with a normal intestinal length; in all previously reported cases, as well as in our patient, no symptoms related to the IC valve's loss were observed. As Iwanaka et al. reported in 1993 that after ileocecal resection without extensive ileal resection in neonates and infants, adequate nutritional status can be maintained [8]. But in 2011, Folaranmi et al. sustained that the loss of the ileocecal valve is complicated by chronic diarrhea [9]. In this series, the age of population is varied and is not clear if the diarrhea was age-surgery related. As Catalano et al. reported in 2014, cyst enucleation together with the common ileal wall, followed by enterorrhaphy, is a feasible and safe alternative to IC resection with primary anastomosis, thus avoiding all clinical problems related to IC valve's loss [10]. Owing to the tight connection of the cyst with the IC valve, the most prudent course of action was an ileocecal resection in order to avoid the risk of stenosis. Despite their rarity in infancy, the surgeon must always be aware of the presence of duplication cysts as a pathologic lead point of intussusception or small bowel obstruction. The early age and the clinical history should be the main features which must raise suspicion of the existence of the pathological lead point. In infants presenting with intussusception without other concomitant factors or with a history of recurrent abdominal distension, additional diagnostic test is always recommended.
